# Association of overweight, obesity and risk of urinary incontinence in middle-aged and older women: a meta epidemiology study

**DOI:** 10.3389/fendo.2023.1220551

**Published:** 2023-10-10

**Authors:** Xin Shang, Yu Fu, Xiaoqin Jin, Chenxiao Wang, Ping Wang, Panpan Guo, Ying Wang, Shuxun Yan

**Affiliations:** ^1^ First School of Clinical Medicine, Henan University of Chinese Medicine, Zhengzhou, China; ^2^ Department of Endocrinology, The First Affiliated Hospital of Henan University of Chinese Medicine, Zhengzhou, China; ^3^ Department of Geriatrics, First Affiliated Hospital of Zhengzhou University, Zhengzhou, China

**Keywords:** overweight, obesity, urinary Incontinence, meta-analysis summary, systematic review

## Abstract

**Aims:**

The aim of this meta-analysis is to evaluate the potential correlation between obesity and overweight, and the vulnerability to urinary incontinence (UI) in women aged middle-aged and above.

**Methods:**

We searched PubMed, Cochrane Library, and Embase for observational studies published between the inception of the databases and April 25, 2023. A fixed-effects model was used when the P>0.1 and the I^2^ ≤ 50%. In cases where I^2^ ≥ 50% (indicating significant heterogeneity), a random-effects model was applied. For the purpose of evaluating publication bias, a funnel plot and Egger’s test were used. Stata 14.0 was used for all statistical analyses.

**Findings:**

This meta-analysis includes 16 observational studies, covering29,618 individuals. The pooled analysis shows that being overweight(25 kg/m^2^≤BMI<30kg/m^2^) in middle-aged and elderly women is more likely to develop UI (OR=1.27; 95% CI: 1.17-1.37; I^2^ = 51.8%, P=0.013). Middle-aged and elderly women with obesity(30 kg/m^2^≤BMI<35 kg/m^2^) are significantly more likely to develop UI (OR=1.60; 95% CI: 1.42-1.81; I^2^ = 71.8%, P=0.000). In addition, the results indicated a higher probability of UI in middle-aged and older women with obesity class II (BMI≥35 kg/m^2^) (OR=1.85; 95% CI: 1.59-2.16; I^2 = ^48.1%, P=0.103). In subgroup analysis, there is no direct relationship between the obesity in middle-aged and elderly women and an increased risk of stress urinary incontinence (SUI) (OR=1.31; 95% CI: 0.99-1.74; I^2^ = 63.7%, P=0.011). In middle-aged and elderly women with obesity are more likely to develop urgent urinary incontinence (UUI) (OR=2.11; 95% CI: 1.54-2.89; I^2^ = 80.2%, P=0.000).

**Conclusion:**

In this meta-analysis, overweight and obesity are associated with an increased risk of UI in middle-aged and elderly women. Obesity and overweight are independent risk factors for UI, as demonstrated by this study.

**Systematic Review Registration:**

https://www.crd.york.ac.uk/PROSPERO/, identifier CRD42023421986.

## 1 Introduction

Urinary incontinence (UI) refers to the involuntary loss of urine from the urethra, which is not controlled by the individual’s will (1). It is a common yet often neglected condition, with an estimated incidence rate ranging from 10% to 40% (2). There are three types of UI: stress urinary incontinence (SUI), urgency urinary incontinence (UUI) and mixed urinary incontinence (MUI) (3).

It is widely believed that the mode and frequency of delivery are commonly considered the main causes of female UI (4). However, the prevalence rate of UI in women of all ages is expanding, particularly with the emergence of social aging (5, 6). The issue is more pronounced in middle-aged and elderly women (7). Although UI affects a majority of women, it is often a private issue that prevents them from actively seeking timely treatment, resulting in potential long-term consequences. Therefore, early identification of risk factors linked to UI could aid in preventing its onset and progression.

Recent studies have demonstrated that overweight and obesity pose a heightened risk for UI in women (8–10). The prevalence of obesity globally is highest in the age range of 60 to 74 years, with roughly 48% of women in the 50-54 age group being overweight and approximately 19% being obese (11). Notably, severe obesity (defined as BMI ≥ 35 kg/m^2^) is markedly more common in women compared to men (12). Additionally, overweight and obesity have been identified as robust predictors of UI, with the risk being significantly elevated in cases of obesity. Nevertheless, no published meta-analysis currently exists that comprehensively examines the relationship between overweight/obesity and UI risk in middle-aged and elderly women. The purpose of this study is to (i) examine the association between overweight, obesity, and UI in middle-aged and elderly women, and (ii) obesity is investigated in relation to UI subtypes.

## 2 Methods

This investigation adhered to the Preferred Reporting Items for Systematic Reviews and Meta-Analyses (PRISMA) guidelines (13) and the protocol was preregistered on the PROSPERO platform (CRD42023421986), exhibiting its commitment to transparent and thorough methodology.

### 2.1 Data sources

A systematic search of relevant databases, including PubMed, Cochrane Library, and EMBASE, was conducted up until 25 April 2023. Medical subject headings (MeSHs) and keywords were combined in the search strategy, with no language restrictions. The keywords used were “obesity”, “obese”, “urinary incontinence”, “uroclepsia”, and “uracratia”, along with their variants (**Appendix, Text S1**). The reference lists of included studies and relevant systematic reviews were also manually searched to identify potentially eligible studies. Our search approach was aimed at ensuring an exhaustive identification of relevant study.

### 2.2 Eligibility criteria

The eligible studies were required to meet the following criteria (1): case-control, cross-sectional, or cohort study design; (2) investigation of the association between obesity, overweight, and the risk of UI; (3) use of adjusted OR and corresponding 95% CI to present the risk of UI as the outcome. In this study, ‘overweight’ was defined as ‘25 kg/m^2^≤BMI ≤ 30kg/m^2^’, ‘obesity’ was defined as ‘BMI ≥30kg/m^2^’and ‘obesity classⅡ’ was defined as ‘BMI ≥35kg/m^2^’.

The analysis excluded trials that did not report an OR estimate along with its 95% CI, in situations where multiple studies presented data from the same cohort, the study with the longest follow-up period or the largest number of participants was selected to ensure the inclusion of the most comprehensive and reliable information in our analysis. In addition, conference abstracts, study protocols, duplicate publications, and studies that did not report outcomes of interest were excluded.

### 2.3 Study selection

Based on predefined eligibility and exclusion criteria, two independent reviewers (X Shang and XQ Jin) carefully screened the literature. Initially, duplicate and irrelevant articles were eliminated based on their titles and abstracts. After obtaining the complete texts of the potentially eligible articles, their eligibility was assessed thoroughly. In case of any discrepancies, discussions were held with SX Yan to arrive at a consensus. The selection of studies was thus meticulously carried out to ensure the reliability and validity of the final results.

### 2.4 Data extraction

X Shang and XQ Jin independently extracted the pertinent data, including the first author, publication year, country or region of origin, study design, sample size, duration of follow-up, study period, age range, diagnostic criteria utilized, and confounder adjustments. The extracted data were subsequently reviewed and verified by SX Yan, who engaged in discussions with the former two researchers in cases where discrepancies arose.

### 2.5 Risk of bias

The quality of cohort studies was assessed using the Newcastle-Ottawa Scale (NOS) (14), which consists of stars ranging from 0 to 9 for selecting participants and measuring exposure, 2 stars for comparing results and 3 stars to the assessment of outcomes and adequacy of follow-up. A higher score indicates a higher quality study. Scores 0-3 indicate low quality, 4-6 indicate moderate quality, and 7-9 indicate high quality. To evaluate the quality of a cross-sectional study, we adopted the American Agency for Health Care Quality and Research’s (AHRQ) (15) quality evaluation items for cross-sectional studies and scored each item as ‘1’ for a ‘YES’ response, or ‘0’ for an ‘UNCLEAR’ or ‘NO’ response. The resulting scores were categorized into low quality (0–3), moderate quality (4–7), and high quality (8–11).

### 2.6 Statistical analysis

Software Stata 14.0 was used for the analysis, whereby adjusted OR and their 95% CI were extracted from the included studies to assess the association between obesity and UI risk. Heterogeneity was evaluated using the χ2-test and I^2^-values (16). In such high-quality research (17), it is mentioned that I^2^ ≤ 50% and P > 0.1, we used a fixed-effects model. Conversely, a random-effects model was adopted if I^2^ exceeded 50%, indicating significant heterogeneity (18). To ensure the validity of the overall effects, sensitivity analysis was performed. Additionally, funnel plots and Egger’s regression test were employed to evaluate for publication biases (19).

## 3 Results

### 3.1 Literature selection

**Figure 1:**
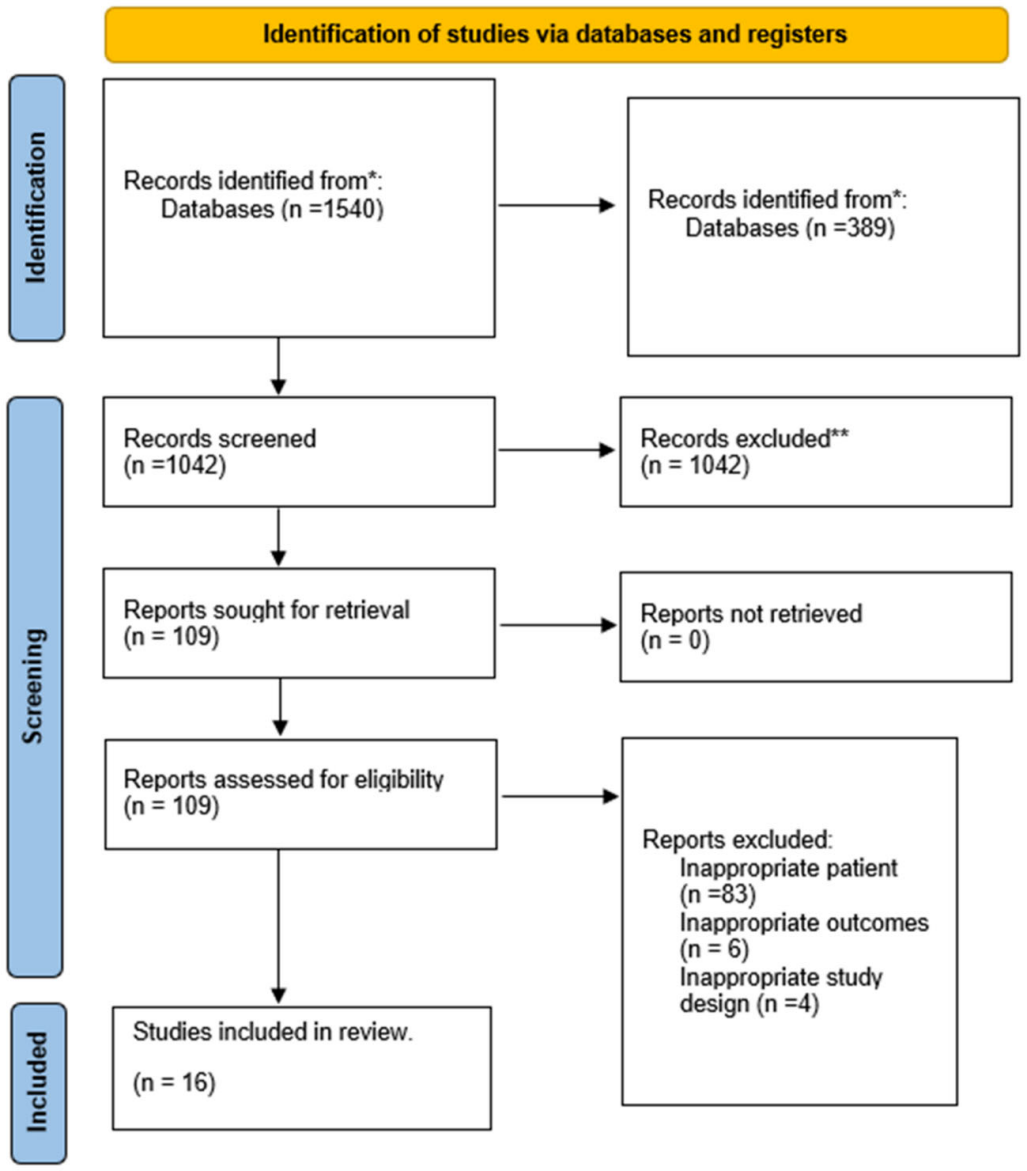
Literature screening flowchart.

A systematic search was conducted for observational studies published before April 25, 2023, yielding a total of 1,540 results. Subsequently, 389 duplicate articles were removed, and 1,042 articles were excluded following the screening of titles and abstracts. After reading the full-text, 109 articles were further excluded, with 92 identified as inappropriate due to patient population, three due to inappropriate outcomes, and four due to inappropriate study design. Ultimately, a total of 16 studies were included in this systematic review. Details of the selection process are presented in **Figure 1**.

### 3.2 Study characteristics

This meta-analysis encompasses a total of 16 studies (20–35),which aimed at investigating an overall sample size of 27,389 individuals. Out of the 16 studies, eleven were cohort studies (20–22, 24, 25, 30–35), while the remaining five were cross-sectional studies16,19-22 (23, 26–29). The studies included in this analysis were published between 2007 and 2023. The sample size of the studies ranged from 168 to 8,736 participants. The majority of the studies presented adjusted estimates, however, some studies did not, including seven studies (22, 24–28, 32).The adjusted confounders used in these studies varied slightly. Detailed information of the characteristics of these 16 studies are provided in **Table 1**.

**Table 1:** Basic characteristics of the included studies.

Author	Year	Country	Study Type	Follow-up years	Diagnostic criteria	Sample size	Age (Mean ± SD)	Confounders adjusted	Scores
Townsend MK, et al. (21),	2008	USA	cohort study	2	self-reported	Total: 6,790 obesity: 3,740 No obesity: 3,050	66 54–79	age, parity, race/ethnicity, smoking, menopausal status, hysterectomy, physical activity, diabetes, high blood pressure, and diuretic use.	7
Martinez A, et al (22)	2022	US	cohort study	20	self-reported	Total: 307	72.7 ± 6.1 ≥65	NA	6
Sievert LL, et al (23)	2022	the State of Qatar.	cross-sectional study	NR	self-reported	Total: 304	49.29 ± 5.55 40-60	menopausal status, level of education, and parity.	6
McGrother CW, et al. (2015),	2015	UK	cohort study	1	self-reported	Total: 837	59.5 ± 13.0 40-98	NA	5
Byles J, et al. (25),	2009	Australia	cohort study	9	self-reported	NA	70–75	NA	6
Islam RM, et al. (26),	2018	Bangladeshi	cross-sectional study	NR	self-reported	Total: 377	42.3 ± 8.1 30-59	NA	5
Zeleke BM, et al. (27),	2015	Australia	cross-sectional study	NR	self-reported	Total: 549 obesity: 394 No obesity: 153	71.5 ± 4.1 65-79	NA	5
Hakimi S, et al. (28),	2020	Iran	cross-sectional study	NR	self-reported	Total: 168	54.3 ± 4.0 45-60	NA	6
Dellú MC, et al (29)	2016	São Paulo	cross-sectional study	NR	self-reported	Total: 204	51.9 ± 8.8 35-72	Menopause, UI pregnancy, UI post-partum, Genital prolapse, Stress, Depression, Obesity	5
Mishra GD, et al (30)	2008	UK.	cohort study.	7	self-reported	NA	48-54	age, childhood enuresis, kidney infection, childbirth characteristics, menopausal status, general practitioner consultations and educational qualifications	6
Townsend MK, et al. (31),	2007	America	cohort study.	14	self-reported	Total: 4,215 obesity: 2,172 No obesity: 2,043	37-54	age, parity, race or ethnicity, smoking, postmenopausal hormone therapy status, hysterectomy, oral contraceptive use, physical activity, and diabetes.	7
Mitchell ES, et al. (32),	2013	Seattle	cohort study	20	self-reported	NA	41.5 ± 4.3 35-55	NA	6
Forsman M, et al. (33),	2008	Sweden.	cohort study	32	self-reported	Total: 555	64.1 ± 9.2 48-81	age,BMI and childbirth (ever/never)	8
Janssen I, et al. (34),	2007	US	cohort study	9	self-reported	Total: 2,001	≥65 years	age, sex, race, socioeconomic status, smoking, and physical activity.	7
Komesu YM, et al. (20),	2011	US	cohort study	2	self-reported	Total: 2,286	67.6 ± 10.15 ≥50	age, ethnicity, parity, medical comorbidities, functional limitations, psychiatric illness, BMI, and UUI and SUI status at baseline	7
Hjorth S, et al. (35),	2023	Denmark	cohort study	7	self-reported	Total: 8,736 obesity: 3,033 No obesity: 5,703	37.7 ± 4.2	age, number of cesarean sections, number of vaginal births, chronic illness, smoking, and alcohol use at 7‐year follow‐up, plus socioeconomic position, and physical activity at the index pregnancy.	6

### 3.3 Quality assessment

We assessed the quality of the eighteen studies via the NOS and AHRQ, and the scores are presented in **Table 1**. Five studies (20, 21, 31, 33, 34) were scored as ≥7 (high quality) and six studies (22, 24, 25, 30, 32, 35) were scored as 6 and 5 (moderate quality) according to the NOS criteria. An average score of 6.5 was obtained from these thirteen studies, representing an overall moderate quality. Five studies (23, 26–29) were scored as 6 and 5 (moderate quality) according to the AHRQ criteria. The average score of these five studies was 5.4, representing an overall moderate quality.

### 3.4 Overweight and risk of UI

**Figure 2:**
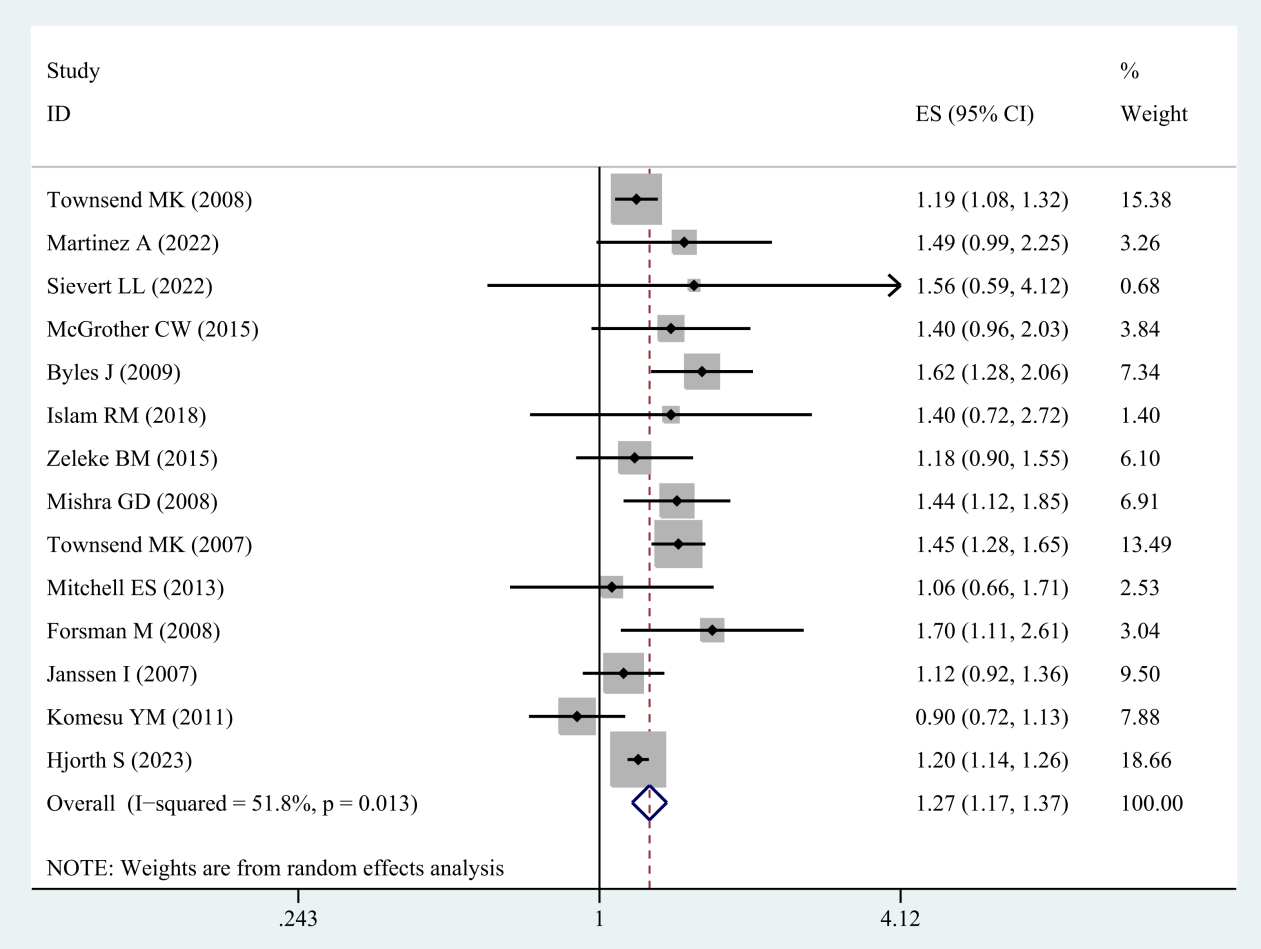
Meta-analysis of overweight and the risk of UI in middle-aged and older women.

A total of 14 studies (20–27, 30–35) examined the relationship between overweight and UI in middle-aged and older women. The synthesis of these studies reveals that overweight is significantly associated with a heightened risk of UI (OR= 1.27; 95% CI: 1.17-1.37; I^2^ = 51.8%, P=0.013; **Figure 2**).

Furthermore, the sensitivity analysis of the individual studies did not demonstrate a reversal of the pooled-effect size, indicating the robustness of the results (**Supplementary Figure A**).

### 3.5 Obesity and risk of UI

**Figure 3:**
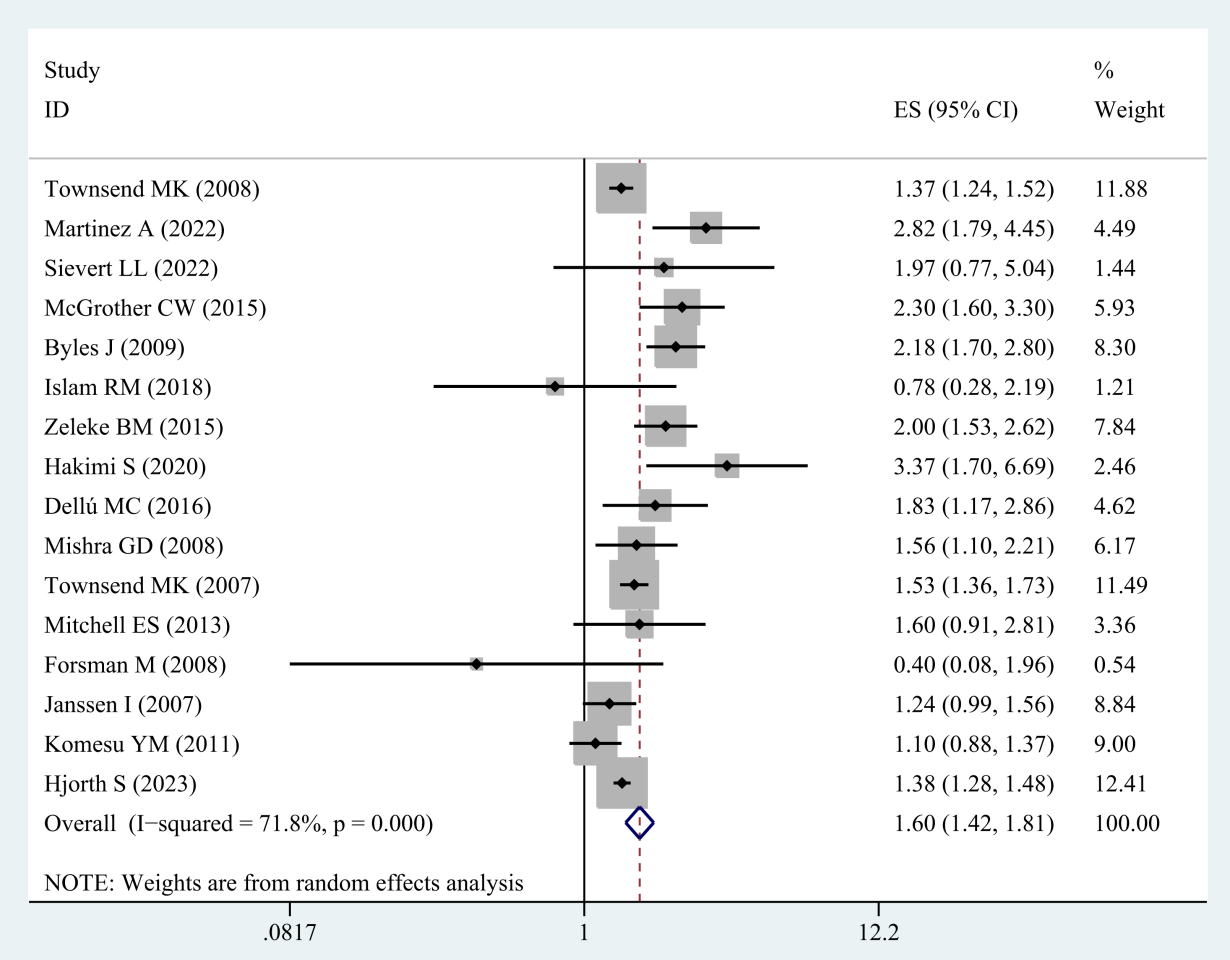
Meta-analysis of obesity and the risk of UI in middle-aged and older women.

Sixteen studies (20–35) were included in the analysis evaluating the correlation between obesity and the risk of UI among middle-aged and elderly women. There is a significant association between obesity and UI in this group of women (OR = 1.60; 95% CI: 1.42-1.81; I^2^ = 71.8%, p=0.000; **Figure 3**).

Moreover, the sensitivity analysis corroborated the robustness of the results, as none of the individual studies changed the pooled effect size (**Supplementary Figure B**).

### 3.6 Obesity class II and risk of UI

**Figure 4:**
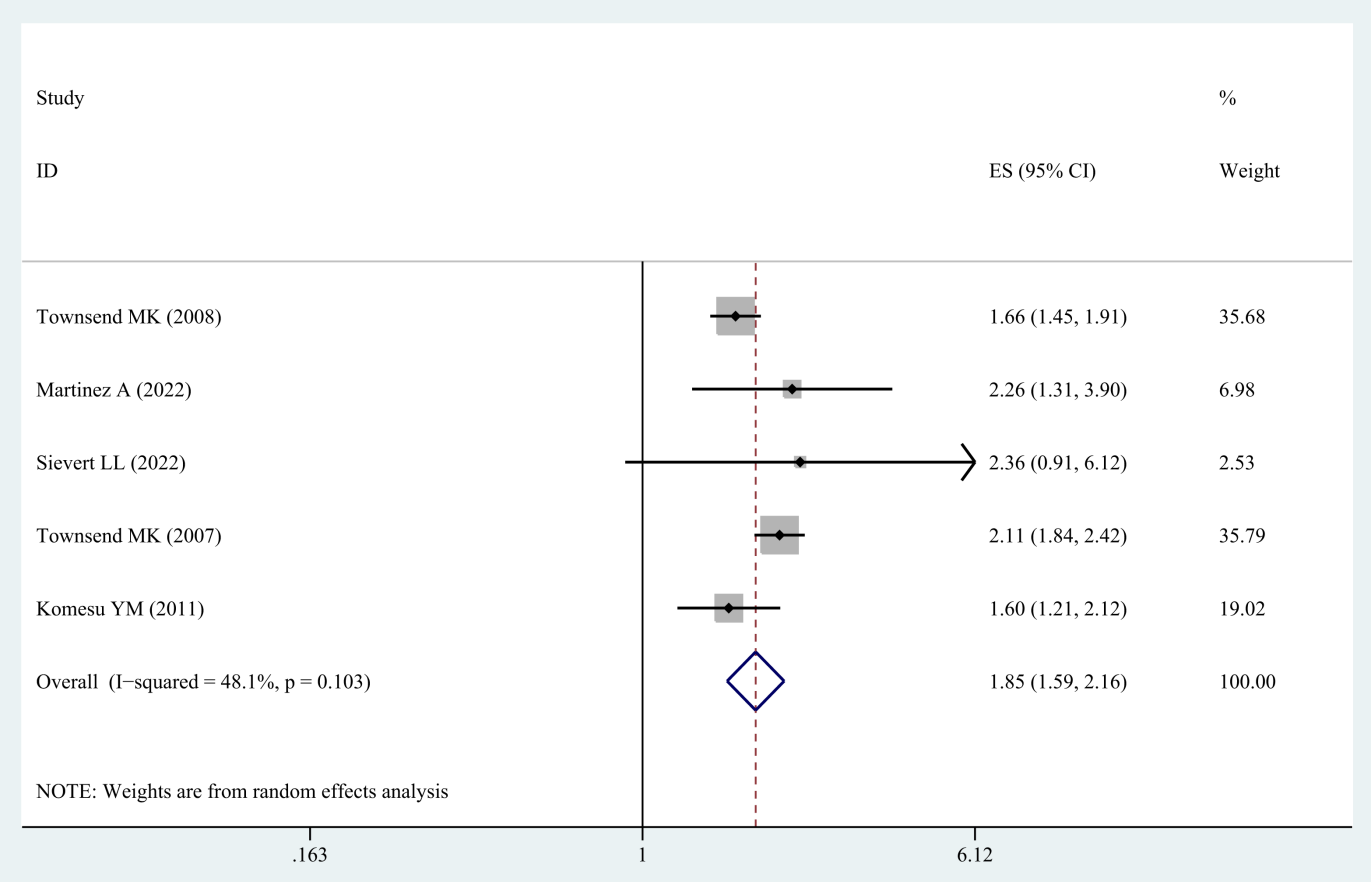
Meta-analysis of obesity class II and the risk of UI in middle-aged and older women.

Five studies (20–23, 31) were incorporated in order to examine the correlation between obesity class II in middle-aged and older women and the possibility of UI. In general, the results indicated a higher probability of UI in middle-aged and older women with obesity class II (OR=1.85; 95% CI: 1.59-2.16; I^2^ = 48.1%, p=0.103; **Figure 4**).

According to the sensitivity analysis, none of the included studies significantly reversed the pooled effect size when analyzed individually. This indicates that the results obtained from the pooled analysis are robust and reliable (**Supplementary Figure C**).

### 3.7 Subgroup analysis

We performed a subgroup analysis on the various types of UI in our study. The results indicated no significant association between obesity and stress urinary incontinence (SUI) in middle-aged and elderly women (OR=1.31; 95% CI: 0.99-1.74; I^2^ = 63.7%, P=0.011). Our analysis, however, revealed that obesity is significantly associated with urge urinary incontinence (UUI) (OR=2.11; 95% CI: 1.54-2.89; I^2^ = 80.2%, P=0.000) (**Table 2**).

**Table 2:** Subgroup analysis for the risk of obesity in middle-aged and elderly women with UI.

Subgroups	Included studies	OR	Heterogeneity	
		95% CI	I^2^ (%)	P-values
UI type				
SUI	7	1.31(0.99-1.74)	63.70%	0.011
UUI	8	2.11(1.54-2.89)	80.20%	0.000

### 3.8 Publication bias

**Figure 5:**
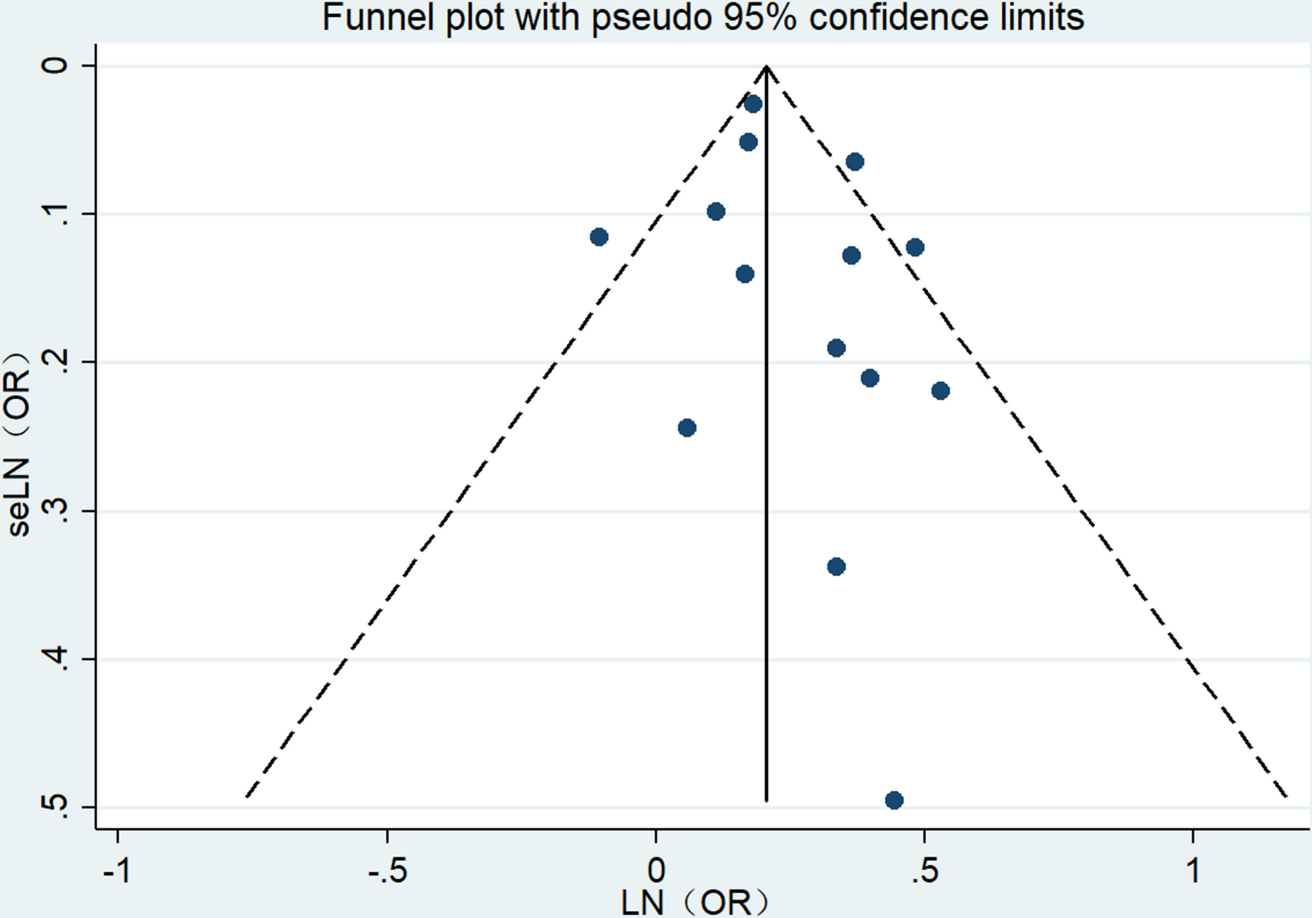
Funnel plot.

The visual inspection of the funnel plot in our meta-analysis revealed a notable absence of any significant publication bias concerning the relationship between obesity in individuals of middle and advanced age and the risk of UI (**Figure 5**). Moreover, Egger’s regression test (P = 0.294) confirmed that our meta-analysis did not contain any publication bias.

## 4 Discussion

### 4.1 Main findings

The present meta-analysis involved a total of 16 studies that encompassed a vast sample of 27,389 individuals, and thoroughly assessed the association between overweight, obesity, and the likelihood of UI in middle-aged and elderly women. Our systematic analysis revealed that the risk of developing UI in overweight and obese middle-aged and elderly women was significantly higher, with an overall risk increase of 1.27-fold, 1.60-fold, and 1.85-fold, respectively, compared to the non-obese control group. These results suggest that both overweight and obesity may potentiate the risk of UI as independent contributory factors.

### 4.2 Interpretation of findings

It is noteworthy that a previous review has explored the correlation between overweight and obesity and UI in young to middle-aged women, confirming them as risk factors for UI (8). However, the health concerns of middle-aged and elderly women have become increasingly important with a growing aging population. Therefore, our study aims to examine the association between overweight and obesity in middle-aged and elderly women and UI, providing more robust evidence through a greater number of cohort and cross-sectional studies. We also conducted a subgroup analysis to examine the impact of overweight and obesity on different types of UI, enhancing our understanding of their specific effects. Our study results highlight overweight and obesity as significant risk factors for UI in middle-aged and elderly women, emphasizing the need for their consideration in clinical practice.

An alternative investigation examined a meta-analysis pertaining to BMI, abdominal obesity, weight gain, and their association with UI risk (10). The findings demonstrated that the total RR for each 5 kg/m^2^ increase in BMI was 1.20 (95% CI: 1.16-1.25, I^2^ = 58%, n=13), whereas the RR for every 10 kg weight gain was 1.34 (95% CI: 1.11-1.62, I^2^ = 90%, n=2). Notably, no correlation was established between high BMI and UI risk in middle-aged and elderly women. Hence, we sought to re-examine and analyze the link between overweight/obesity and UI risk in middle-aged and elderly women. Our study revealed that both overweight and obesity augment UI risk in this demographic.

The global prevalence of UI has reached 8.7%. Studies indicate that approximately 50% of adult women may experience UI, which is twice the incidence rate of male UI (36, 37). The likelihood of developing UI increases with age and it is a common condition among the elderly. This condition has a considerable impact on quality of life, as it can hinder normal social activities and physical health (38). Although UI is strongly associated with age, recent research suggests that obesity plays a significant role in both the prevalence and 5-year incidence rate of UI (39). Overweight and obesity have been identified as risk factors for UI in women, with obese women being more susceptible to this condition than their normal-weight counterparts. However, limited research is available on the pathophysiological mechanisms underlying the association between obesity and UI.

Research has revealed that middle-aged and elderly women face a significantly higher risk of UI as they age (35, 40). Many women attribute UI to age and underestimate the impact of overweight and obesity on their symptoms, presuming it to be a natural occurrence. Due to several factors, women often conceal their UI medical history, and even when diagnosed, few seek active treatment (41, 42). However, the impact of obesity on the UI of middle-aged and elderly women should be given more consideration. Obesity has also been linked to UI surgery in certain studies. Weight loss interventions are beneficial in improving UI, and procedures like weight loss surgery can ameliorate the quality of life of UI patients (43, 44).

The female urinary system has a unique structure that increases the susceptibility to risk factors such as pregnancy, childbirth, and hysterectomy, which might damage pelvic floor muscle and connective tissues. In postmenopausal women, with decreased estrogen levels, there is a reduction in the capacity to maintain the health of the bladder lining and urethra, leading to tissue degradation and exacerbation of UI development. Furthermore, with increasing age, the muscles of the bladder and urethra become weak and lose their contractility. decreased bladder capacity and feeling of fullness, decreased rate of detrusor muscle contraction, decreased pelvic floor muscle resistance and increased residual urine volume. Dysfunction of the detrusor and sphincter reduces bladder capacity, thereby increasing the risk of involuntary urination (45). Overweight and obesity can impair normal urination and elevate the incidence of UI. Being overweight places more stress on the bladder and surrounding muscles, leading to a decrease in their strength and urine leakage during coughing or sneezing. Central adipose tissue weight may result in a chronic increase in abdominal pressure, causing tension in the urethral support, increasing the risk of UI development. Apart from these reasons, an increase in BMI might trigger UI development via other mechanisms. Overweight and obesity result in oxidative strain and chronic insulin resistance, leading to pelvic floor vascular damage and sclerosis of detrusor and sphincter. Current research conducted in high-income countries suggests that SUI is the most prevalent type of UI (6, 46). Our research also supports the argument that BMI is an independent predictor of SUI.

The development of UI may pose a series of associated challenges, including persistent skin moisture leading to skin problems such as rashes, infections, and sores, an increased risk of recurrent urinary tract infections, as well as physical and psychological discomfort that can disrupt personal lives. Recent studies indicate that a longer period of being overweight or obese heightens the risk of UI, thus calling for targeted interventions aimed at enhancing physical health among middle-aged and elderly women to prevent UI. Our findings reveal a strong association between high BMI, abdominal pressure, and bladder pressure, lending credence to the proposition that avoiding high BMI may help alleviate the burden of UI reported among middle-aged and elderly women.

### 4.3 Implications and limitations

This meta-analysis provides a comprehensive overview of the current evidence concerning the association between overweight/obesity in middle-aged and elderly populations and the risk of UI. Our findings highlight the necessity of increased attention towards the risk of UI in obese patients during these life stages, facilitating the early identification of high-risk populations for UI.

It is important to note, however, that our study has certain limitations. First and foremost, only 16 related studies were eligible for inclusion due to the complexity of grouping types. Moreover, subgroup analysis was solely performed on two common subtypes of SUI and UUI, covariate analysis was not conducted. The adjustment factors incorporated into the study exhibit variability, with certain studies omitting any form of adjustment. This divergence in adjustment practices could potentially impact the precision of the findings. Nonetheless, most of the included studies mentioned adjusted confounding factors, effectively controlling for confounding bias. Therefore, our results are convincing with considerable clinical practicality.

Due to the absence of standardized diagnostic criteria, we have synthesized several definitions of UI in our analysis. The diagnostic criteria for UI vary across different questionnaires and can be broadly categorized into three groups. First, UI is defined based on the criterion of experiencing leakage at least once a month or less frequently, with enough fluid discharge to wet the inner clothing. Second, definitions are established according to the frequency of UI occurrences within the past year. Third, some questionnaires specifically highlight the origin of their criteria, employing established tools like Questionnaire for Urinary Incontinence Diagnosis (QUID) or International Consultation on Incontinence Questionnaire (ICIQ) to delineate UI. Moreover, SUI is defined as instances of urinary leakage during activities such as coughing, sneezing, exercising, or laughing.

Lastly, all included studies relied on questionnaires and self-reports to ascertain UI diagnosis, which may lead to high-risk respondent bias in observational studies and an increased rate of misdiagnosis. The results obtained from self-reported questionnaires are subjective and cannot be considered equivalent to clinical diagnoses. The inherent inaccuracies of self-reporting measurements can compromise the accuracy of our risk assessment. While these findings might align with those of other studies, it is essential to conduct further research involving larger and more diverse populations across various age groups. It is worth noting that previous studies have validated the reliability and effectiveness of self-reporting UI, indicating that it is not a significant limitation (47). Self-reported UI displays an 83% concordance rate with clinician assessments (48). Thus, clinicians should focus on the thorough examination, diagnosis, and treatment of UI patients.

## 5 Conclusions

Among women in middle and old age who experience UI, this is the primary meta-analysis to examine the correlation between overweight and obesity. Overweight and obesity increase UI risk during this stage of life. Furthermore, the risk for various subtypes of UI persists and magnifies in women with a BMI exceeding 30. Nevertheless, A deeper understanding of the pathophysiology of this phenomenon is required. Therefore, healthcare professionals and researchers should contemplate preventing weight gain as a prophylactic measure against UI, and develop incontinence management strategies for obese women in middle and old age.

## Data availability statement

The original contributions presented in the study are included in the article/**Supplementary Material**. Further inquiries can be directed to the corresponding authors.

## Author contributions

XS and SY conceived the study. XS and XJ collected the data and drafted the manuscript. SY revised the manuscript and language. All authors contributed to the article and approved the submitted version.
