# Supplementary material for: Association of overweight, obesity and risk of urinary incontinence in middle-aged and older women: a meta epidemiology study
Source: Front Endocrinol (Lausanne). 2023 Oct 10;14:1220551. doi: 10.3389/fendo.2023.1220551 (PMC10598345; doi:10.3389/fendo.2023.1220551)
Supplement: Supplementary file 1 [file DataSheet_1.docx]

Supplementary Material

Association of Overweight, Obesity and Risk of Urinary Incontinence in Middle-Aged and Older Women: A Meta Epidemiology Study

**Xin Shang 1†, Yu Fu 2†, Xiaoqin Jin2, Chenxiao Wang2, Ping Wang1, Ying Wang 3* and Shuxun Yan 2***

*** Correspondence:** Shuxun Yan [ysx982001@163.com](mailto:ysx982001@163.com)Ying Wang [wangying6662001@hotmail.com](mailto:wangying6662001@hotmail.com)

**The retrieval strategies and retrieval results of each database are shown in Tables 1-3**.

**Table 1** **|** PubMed

| No. | Content | Result |
| --- | --- | --- |
| #1 | Search: "Obesity"[Mesh] Sort by: Most Recent | 257,334 |
| #2 | Search: (obesity[Title/Abstract]) OR (obese[Title/Abstract]) Sort by: Most Recent | 377,958 |
| #3 | Search: "Overweight"[Mesh] Sort by: Most Recent | 268,570 |
| #4 | Search: overweight[Title/Abstract] Sort by: Most Recent | 87,303 |
| #5 | Search: ((("Obesity"[Mesh]) OR ((obesity[Title/Abstract]) OR (obese[Title/Abstract]))) OR ("Overweight"[Mesh])) OR (overweight[Title/Abstract]) Sort by: Most Recent | 448,361 |
| #6 | Search: "Urinary Incontinence"[Mesh] Sort by: Most Recent | 36,006 |
| #7 | Search: ((Urinary Incontinence[Title/Abstract]) OR (Uroclepsia[Title/Abstract])) OR (Uracratia[Title/Abstract]) Sort by: Most Recent | 28,466 |
| #8 | Search: ("Urinary Incontinence"[Mesh]) OR (((Urinary Incontinence[Title/Abstract]) OR (Uroclepsia[Title/Abstract])) OR (Uracratia[Title/Abstract])) Sort by: Most Recent | 45,259 |
| #9 | Search: (((("Obesity"[Mesh]) OR ((obesity[Title/Abstract]) OR (obese[Title/Abstract]))) OR ("Overweight"[Mesh])) OR (overweight[Title/Abstract])) AND (((Urinary Incontinence[Title/Abstract]) OR (Uroclepsia[Title/Abstract])) OR (Uracratia[Title/Abstract])) Sort by: Most Recent | 953 |

**Table 2 |** Embase

| No. | Content | Result |
| --- | --- | --- |
| #1 | 'obesity'/mj | 212,417 |
| #2 | obesity:ab,ti OR obese:ab,ti | 544,400 |
| #3 | #1 OR #2 | 577,283 |
| #4 | 'urine incontinence'/mj | 20,299 |
| #5 | 'urine incontinence':ab,ti OR uroclepsia:ab,ti OR uracratia:ab,ti | 562 |
| #6 | #4 OR #5 | 20,684 |
| #7 | #3 AND #6 | 443 |

**Table 3 |** Cochran Library

| No. | Content | Result |
| --- | --- | --- |
| #1 | MeSH descriptor: [Obesity] this term only | 17,589 |
| #2 | (Obesity):ti,ab,kw OR (Obese):ti,ab,kw | 52,080 |
| #3 | #1 OR #2 | 52,080 |
| #4 | MeSH descriptor: [Urinary Incontinence] this term only | 1,925 |
| #5 | ("Urinary Incontinence"):ti,ab,kw OR (Uroclepsia):ti,ab,kw OR (Uracratia):ti,ab,kw | 8,398 |
| #6 | #4 OR #5 | 8,398 |
| #7 | #3 AND #6 | 144 |


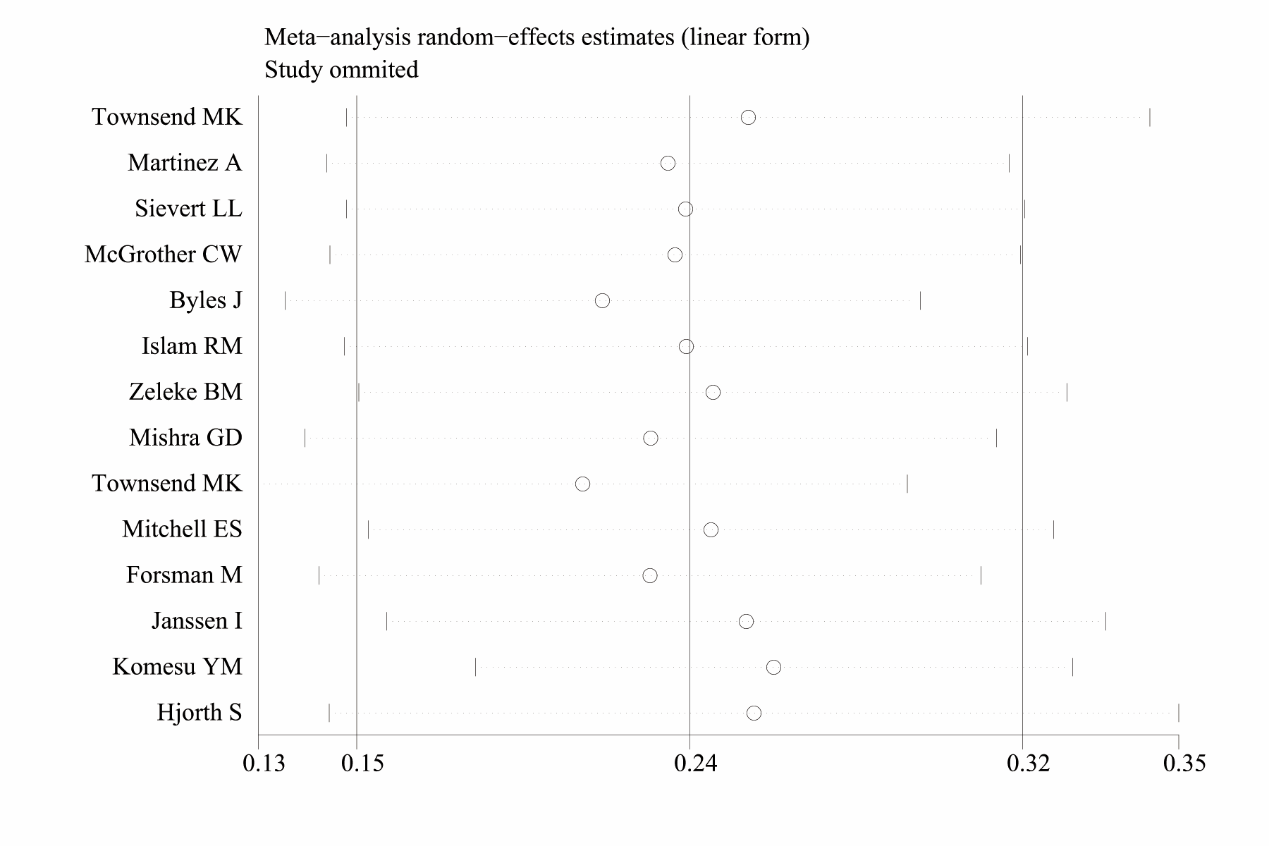


**SUPPLEMENTARY FIGURE A** **| Sensitivity analysis** **of overweight and the risk of UI in middle-aged and older women.**

**
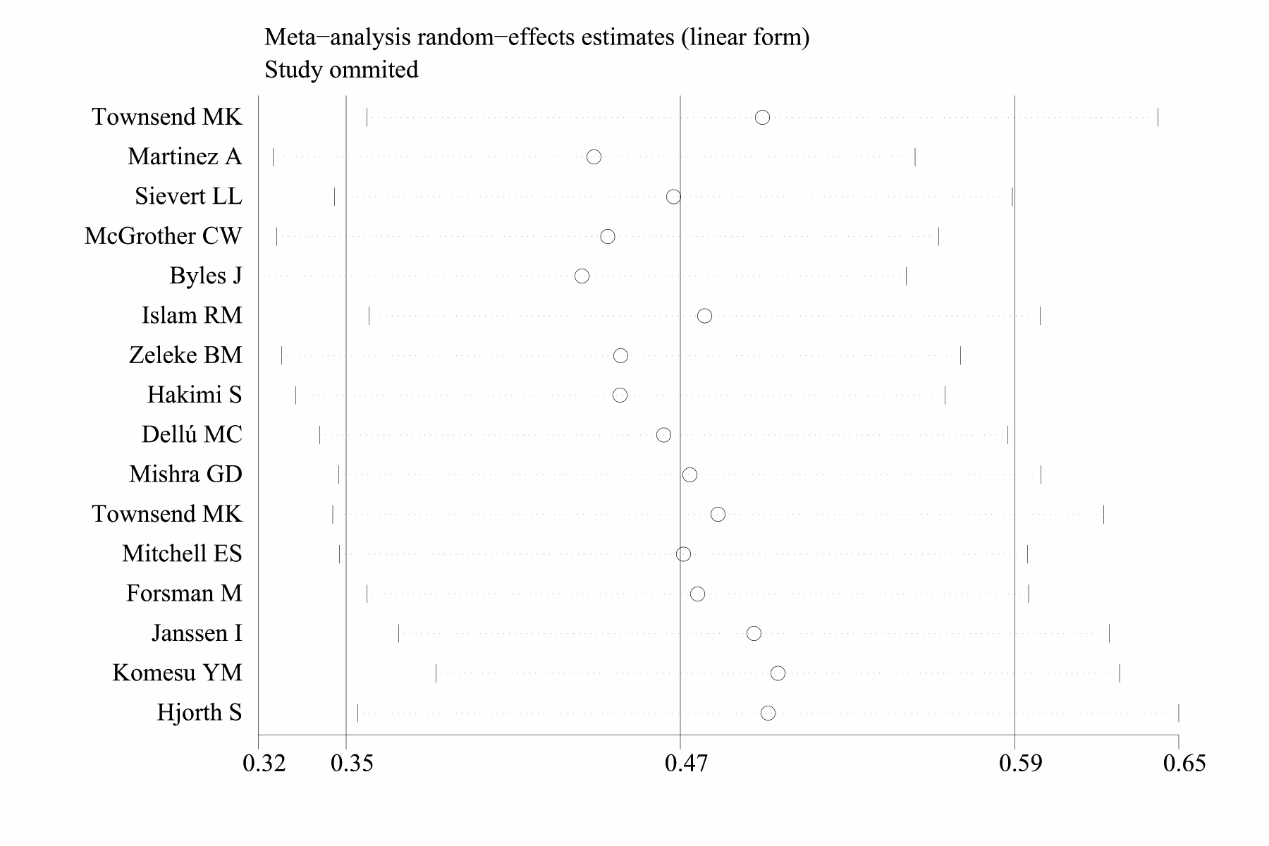
**

**SUPPLEMENTARY FIGURE B** **| Sensitivity analysis** **of obesity and the risk of UI in middle-aged and older women.**

**
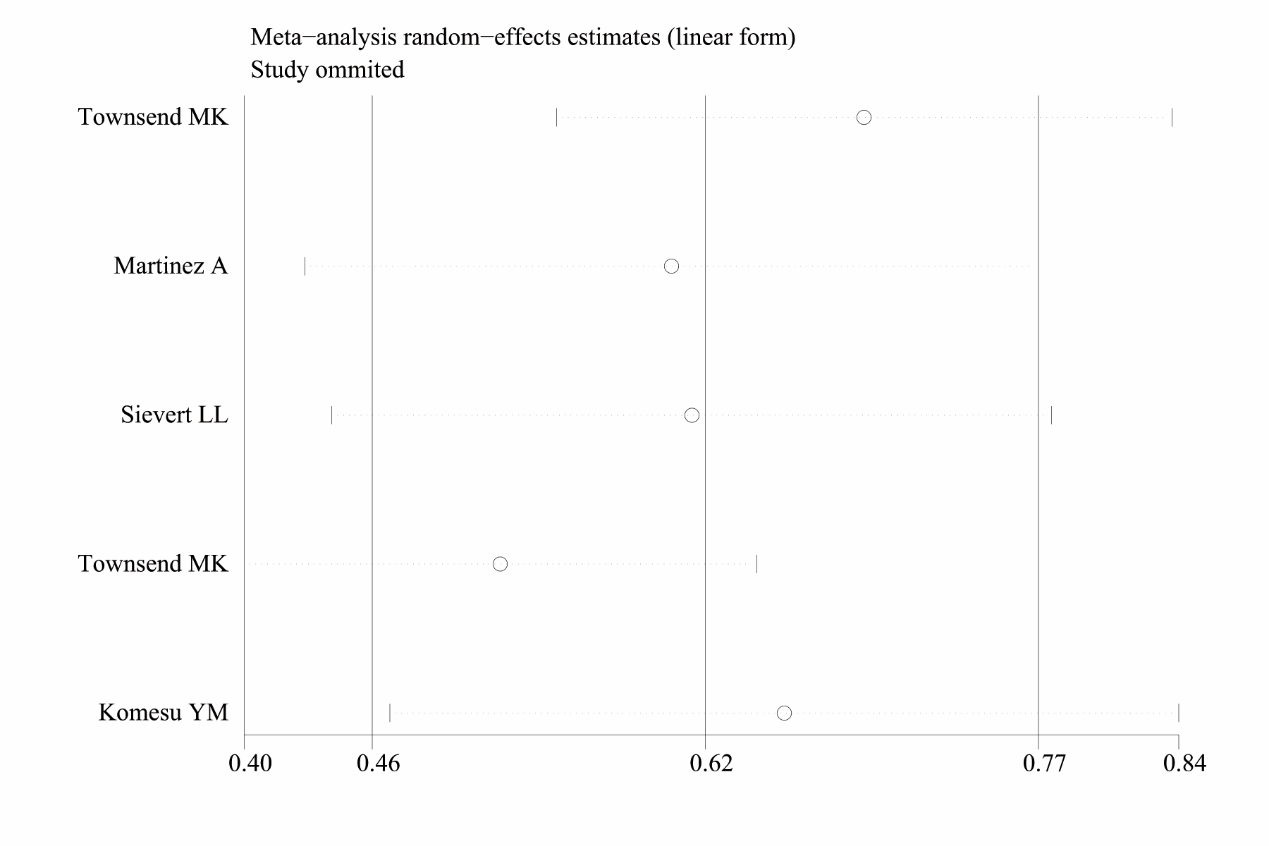
**

**SUPPLEMENTARY FIGURE C** **| Sensitivity analysis** **of obesity classⅡand the risk of UI in middle-aged and older women.**
